# Gaillard’s Medical Journal

**Published:** 1885-12

**Authors:** 


GAILLARD'S MEDICAL JOURNAL.—The February number of this famous magazine will contain a splendid steel engraving of its illustrious founder, the late DR. E. S. GAILLARD.

We are much gratified to learn that the medical profession throughout the United States have not proved recreant in the matter of testifying their admiration for, and appreciation of Dr. Gaillard's genius, and wonderfully versatile talent; but that the patronage and support accorded by them to the *Journal* (which, with his name and fame, are the sole legacies to his family) have been liberal, and such as to elicit a graceful acknowledgement from Mrs. Gaillard, who is personally managing the business. We learn with pleasure that the subscription list has steadily increased during the last twelve months.

We will take this occasion to say that those who do not subscribe for *Gaillard's Journal*, have *no conception* of what they are missing;—it is a *library* in itself, of the choicest reading, fresh and attractive at all times, and fully abreast of the most advanced ideas.

